# Association between semantic dementia and progressive supranuclear palsy

**DOI:** 10.1136/jnnp-2017-317839

**Published:** 2018-04-16

**Authors:** Julie S Snowden, Christopher Kobylecki, Matthew Jones, Jennifer C Thompson, Anna M Richardson, David M A Mann

**Affiliations:** 1 Cerebral Function Unit, Greater Manchester Neuroscience Centre, Salford Royal NHS Foundation Trust, Salford, Greater Manchester, UK; 2 Division of Neuroscience and Experimental Psychology, School of Biological Sciences, University of Manchester, Manchester, UK

**Keywords:** semantic dementia, supranuclear palsy, neuropsychology, neuropathology, clinical neurology

## Introduction

Clinical syndromes associated with frontotemporal lobar degeneration (FTLD) pathology may overlap. Progressive supranuclear palsy syndrome (PSPs) may co-occur with behavioural frontotemporal dementia (bvFTD), non-fluent aphasia (nfPPA) and corticobasal syndrome.^1^ This is unsurprising, given each syndrome’s association with tau pathology. We describe here a less anticipated association: between PSPs and semantic dementia (SD).

## Case history

**Figure 1:**
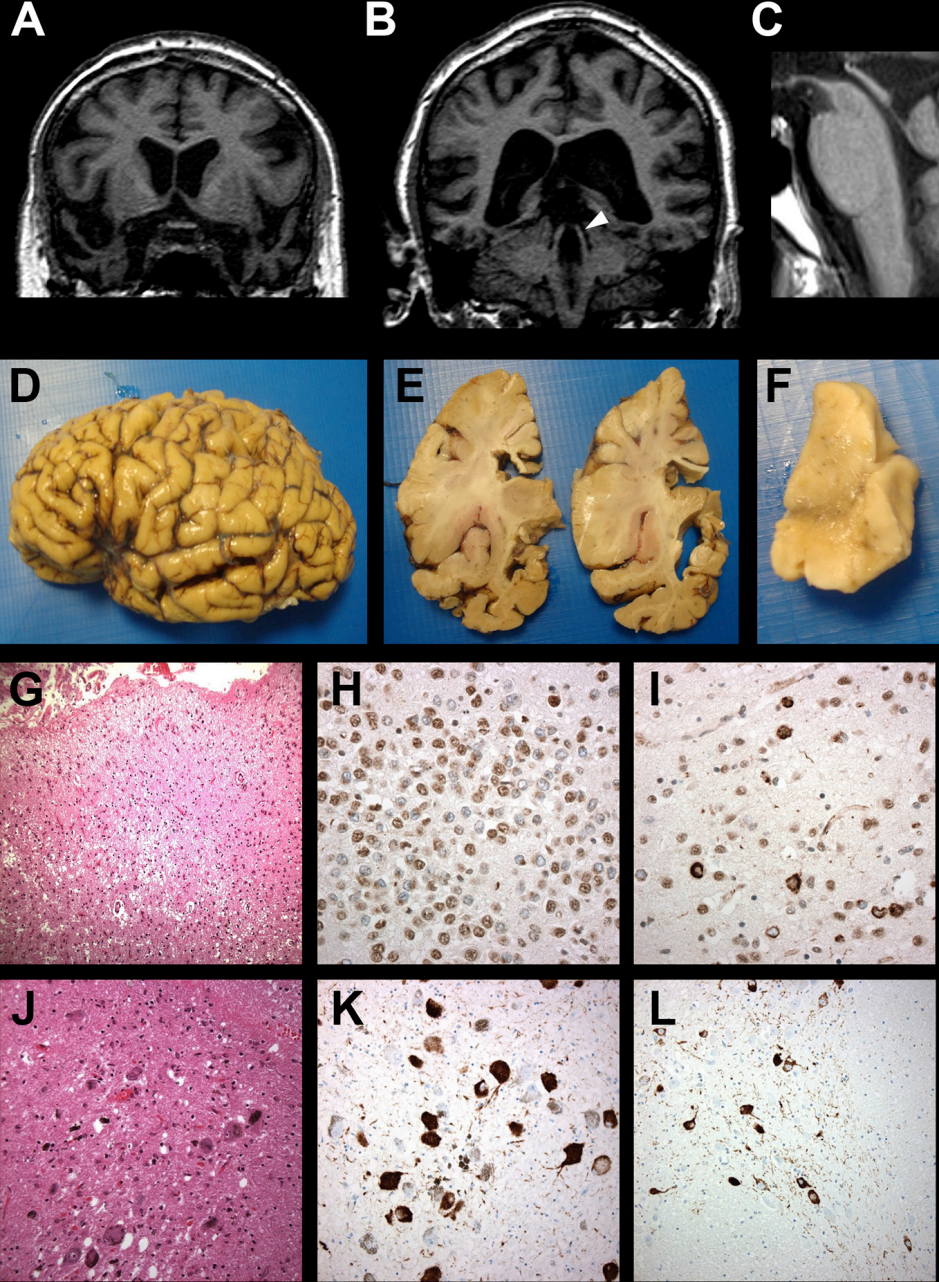
Coronal T1-weighted MR brain imaging showing marked (A) anterior temporal lobe atrophy and (B) atrophic superior cerebellar peduncles (arrowhead). (C) Sagittal T1-weighted image showing midbrain atrophy (midbrain:pons ratio 0.47). Macroscopic image of brain showing temporal lobe atrophy, most marked in inferior temporal gyrus (D, E) and pronounced in the amygdala (E). Substantia nigra shows loss of pigmented neurons (F). Microvacuolation, gliosis and neuronal loss are present in temporal pole (G) and widespread transactive response DNA binding protein 43 (TDP-43) immunoreactive inclusions within dentate gyrus granule cells of hippocampus (H) and pyramidal cells of the temporal cortex (I). Scattered melanophages and pigment incontinence in substantia nigra (J). Globose neurofibrillary tangles, oligodendroglial coiled bodies and neuropil threads are also present in midbrain (K) and basis pontis (L).

A 72-year-old man presented with an 8-year history of difficulty understanding words and phrases and recognising people and places. No behavioural changes were reported. There was no relevant family history. Neurological examination was normal. Neuropsychological examination revealed a severe disorder of semantic, and to a lesser extent, episodic memory. He could not identify high-profile famous faces and names, reporting most to be unfamiliar. He named only 2/30 pictures on the Graded naming test and scored 46/52 and 38/52 on word and picture versions of the Pyramids and Palm trees test. He performed normally on perceptual and spatial tasks (Visual Object and Space Perception Battery), except where recognition of object identity was required. Sentence comprehension (Test of Reception of Grammar) and executive performance (Weigls blocks, Brixton) were preserved. Memory test scores were reduced. However, he was fully oriented in time and place raising the possibility that semantic impairment contributed to his poor scores. An initial MR brain scan showed marked anterior temporal lobe atrophy with right-sided predominance (figure 1A) and atrophy of the superior cerebellar peduncles (figure 1B). The clinical picture suggested SD, although with greater episodic memory loss than commonly found.

Over the following 3 years, he showed a modest decline in semantic and episodic memory, while other cognitive domains remained preserved. No changes in behaviour were reported. In contrast to the slow cognitive progression, there was dramatic physical decline. He became profoundly parkinsonian, with difficulties in initiation of movement, poor balance and frequent falls, invariably backwards. Vertical eye movements were restricted. He was unable to walk unassisted and required a wheelchair. Repeat MR brain imaging showed atrophy of the midbrain (figure 1C). The cognitive profile remained in keeping with SD, whereas the neurological profile was of PSPs. He died at the age of 77, 13 years after symptom onset. Informed consent was given for brain tissue examination.

### Neuropathology

The brain weighed 1179 g and appeared symmetrical. There was marked temporal lobe atrophy, especially anterior inferior temporal gyrus (figure 1D,E) but other cortical regions appeared normal. White matter was preserved throughout. The amygdala was grossly atrophied, hippocampus less so. Corpus callosum was thinned anteriorly. Substantia nigra (figure 1F) and locus coeruleus were both underpigmented.

There was patchy superficial microvacuolation in cerebral cortex, but neuronal loss/gliosis was prominent only in inferior and middle temporal gyri and at temporal pole (figure 1G,J). Sparse flame-shaped neurofibrillary tangles, glial inclusions, neuropil threads and astrocytic plaques were present. There was marked hippocampal sclerosis and amygdala was densely spongiotic with neuronal loss and reactive gliosis, but few tangles. Frequent transactive response DNA binding protein 43 (TDP-43) immunoreactive inclusions were present in the dentate gyrus of the hippocampus (figure 1H), amygdala, entorhinal and temporal cortex, with few short dystrophic neurites (figure 1I). No beta-amyloid or alpha-synuclein pathology was present.

There was severe loss of pigmented neurons from substantia nigra, especially dorsolaterally, with scattered melanophages and pigment incontinence (figure 1J). The midbrain appeared gliotic and globose, tau-positive neurofibrillary tangles, oligodendroglial coiled bodies and neuropil threads were widespread throughout substantia nigra (figure 1K), tectum and tegmentum, locus coeruleus, basis pontis (figure 1L), medullary motor nuclei and inferior olives. Cerebellar white matter showed tau-positive coiled bodies with neuropil threads. Dentate nucleus was gliotic with patchy neuronal loss, globose neurofibrillary tangles and granular pretangles.

There was widespread, patchy vascular hyalinosis with increased perivascular space in basal ganglia and deep white matter of the temporal lobe and cerebellum, but without infarction or haemorrhage.

The apolipoprotein E genotype was E3/E3. Screening for *C9orf72* expansions was negative.

## Discussion

Cognitive change is an integral part of PSPs^1^ and may precede physical symptoms. The present case is, however, unusual. SD is typically associated with TDP-43 and PSPs with tau pathology so, their co-occurrence would not a priori be predicted. To our knowledge, mixed SD/PSPs phenotype has previously been reported only once,^2^ without supportive neuropsychological or pathological data, although impaired semantic test performance has been described.^3^

In the present case, two underlying pathologies were identified, with differing distributions within the brain that mapped, respectively, on to the physical and cognitive disorder: tau pathology conforming to PSP in midbrain structures and TDP-43 in temporal cortex. Interestingly, the TDP-43 pathology, characterised by neuronal cytoplasmic inclusions (NCI), short neuritic profiles, and small, granular NCI in dentate gyrus granule cells, conformed to TDP-43 subtype A,^4^ which is typically associated with nfPPA and some cases of bvFTD. SD is usually associated with type C pathology, consisting of sparse NCI but frequent long, neuritic profiles within temporal cortex, and more rounded, solid NCI, reminiscent of Pick bodies, in dentate gyrus granule cells. Thus, the TDP-43 pathological characteristics differ from those of prototypical SD.

Limited TDP-43 pathology, closely resembling FTLD-type subtype A, is present in many neurodegenerative disorders and has generally been considered a ‘secondary’ change. The present case challenges that assumption. Clinically, the patient’s cognitive and physical disorders showed different trajectories. The semantic disorder emerged early and progressed slowly, whereas physical changes of PSPs developed late and progressed rapidly. Whereas the marked anterior temporal atrophy is typical of SD, atrophy of the superior cerebellar peduncles has been identified as an imaging biomarker for PSP.^5^ The subsequent identification of midbrain atrophy on MRI and pathological examination was also supportive of PSP.^5^ The TDP-43 temporal cortical pathology, sufficient to give rise to clinical symptoms of SD might occur separately and progress independently of other protein aggregations.

In summary, the patient exhibited two distinct clinical syndromes (SD and PSPs), underpinned, respectively, by two different FTLD pathologies (TDP-43 and tau). The different time courses of evolution and progression of symptoms and the different distributions of pathological changes suggest independence of the distinct pathologies. The unusual association between an SD-like cognitive profile and TDP-43 type A, rather than type C, pathology adds further to the complexities of FTLD.
